# Improving mycotoxin enzyme immunoassay performance by optimizing formaldehyde-mediated hapten–protein conjugation: an α-cyclopiazonic acid case study

**DOI:** 10.1016/j.mex.2026.103894

**Published:** 2026-03-31

**Authors:** Leon Hart, Christina Rehagel, Daniela Schale, Madeleine Plötz, Ewald Usleber

**Affiliations:** a Chair of Dairy Sciences, Institute of Veterinary Food Science, Justus-Liebig-University, Giessen, Germany; b Faculty of Food and Nutrition Sciences, Hochschule Niederrhein University of Applied Sciences, Mönchengladbach, Germany; c Institute of Food Quality and Food Safety, University of Veterinary Medicine, Hannover, Germany

**Keywords:** Enzyme immunoassay, Hapten-protein conjugation, Formaldehyde condensation, Assay optimization, Small-molecule immunoassay, Cyclopiazonic acid

## Abstract

Enzyme immunoassays (EIAs) for small molecules often suffer from limited sensitivity or high non-specific background. This study presents a reproducible strategy to improve EIA performance by optimizing hapten–protein conjugation conditions. Using an immunoassay for the mycotoxin α-cyclopiazonic acid (α-CPA) as a case study, key parameters of the formaldehyde-mediated (Mannich) condensation reaction for synthesizing a hapten–bovine serum albumin conjugate were systematically investigated. Variation of reaction temperature (8–37°C) and time (0.25–72 h) showed that short reaction times at low temperature markedly improved assay performance. Under optimized conditions (8°C, 15 min), the detection limit improved from >100 ng/mL to 2.4 ± 0.2 ng/mL, with a strong reduction in non-specific background. The results demonstrate that targeted optimization of hapten–protein conjugation can significantly enhance EIA sensitivity and specificity, which helps to reduce the number of laboratory animals for antibody production. The approach is readily transferable to other indole-containing mycotoxins and was successfully applied to determine α-CPA in fungal agar plugs from mold-ripened cheeses. This study highlights an important aspect of conjugation conditions during immunoassay optimization.

•Systematic optimization of formaldehyde-mediated hapten–protein conjugation for EIAs

•Improved assay sensitivity and background without new antibody production

•Transferable strategy demonstrated for α-CPA analysis in complex fungal samples

## Graphical abstract

**Figure:**
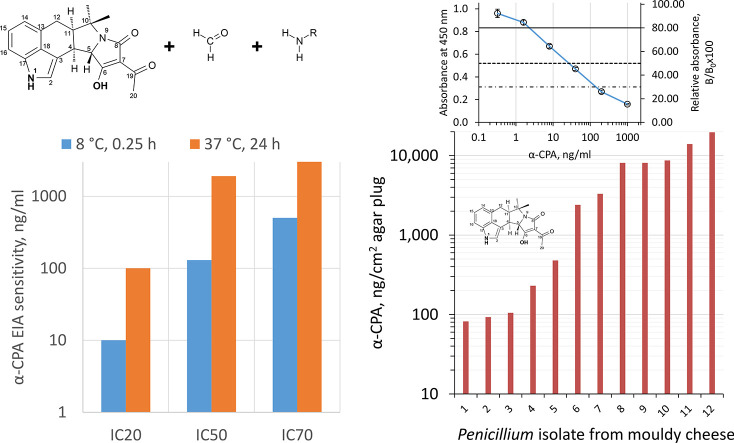

## Specifications table

**Table:** 

Subject area	Biochemistry, Genetics and Molecular Biology
More specific subject area	enzyme immunoassay
Name of your method	formaldehyde-mediated hapten–protein conjugation (Mannich reaction)
Name and reference of original method	Mannich, C.; Krösche, W. Über ein Kondensationsprodukt aus Formaldehyd, Ammoniak und Antipyrin. Archiv der Pharmazie 1912, 250, 647-667. https://doi.org/10.1002/ardp.19122500151

## Background

**Fig. 1:**
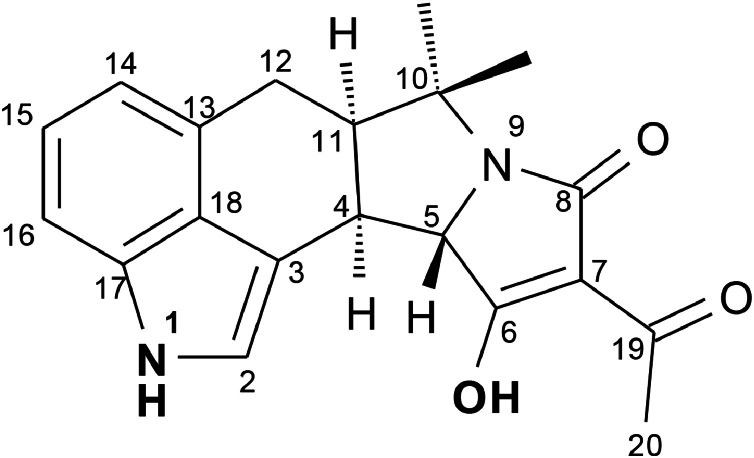
Chemical structure of α-cyclopiazonic acid. The potential reaction sites with formaldehyde are in bold type.

α-Cyclopiazonic acid (α-CPA, Fig. 1) is an indole mycotoxin [1] produced by various species of the genera *Aspergillus* [2] and *Penicillium* [3], including strains relevant in cheese production [4].

Sensitive and reliable analytical methods for small molecules such as α-CPA are desirable, particularly for rapid screening of food and feed. Immunochemical methods, especially enzyme immunoassays (EIAs), represent attractive tools for rapid and cost-efficient analysis. Some EIA methods for α-CPA have been described [[5], [6], [7], [8], [9], [10]]; however, assay performance has been reported to vary considerably, indicating that further optimization of immunoreagents may be required.

A most convenient and easy-to-perform method to produce the immunoreagents necessary for EIA development is formaldehyde condensation, commonly known as the Mannich reaction [11]. Formaldehyde is a simple cross-linking reagent that conjugates amino groups in proteins via methylene linkages. It may also bind haptens via sulfhydryl, phenolic, imidazole, indolyl and guanidinyl groups [12,13]. As stated by Metz et al. [12], the formation of modifications is influenced by various factors, such as the rate of a particular cross-link reaction, the position and local environment of each reactive amino acid in the protein, the pH, the components present in the reaction solution, and the reactant concentrations. The nature of all possible reaction mechanisms of formaldehyde with small molecules other than amino acids has not yet been fully elucidated, particularly with regard to hapten-protein conjugation.

Typically, a 37 % aqueous formaldehyde solution (formalin) is used for cross-linking purposes, in which formaldehyde exists as a series of low-molecular weight polymers that revert to the monomeric form in dilute solutions [13]. The Mannich reaction has successfully been used by our group to conjugate several chemically diverse mycotoxins, such as the hydroxyl-containing compounds citrinin [14] and alternariol [15], as well as the indole-containing ergot alkaloids [16], fumigaclavine A [17], and penitrem A [18]. In these studies, the conditions for conjugation were chosen without systematic optimization of the reaction parameters, mainly due to the relatively limited availability and high costs of commercial mycotoxin standards.

We hypothesize that modification of reaction conditions for hapten-protein conjugation by the Mannich reaction could improve immunoassay performance, specifically assay sensitivity. The aim of this study was therefore to assess the effects of reaction time and reaction temperature on the performance of α-CPA-protein conjugates in competitive indirect EIA, using α-CPA as a representative case study.

The key innovation of this study is the demonstration that systematic optimization of formaldehyde-mediated hapten-protein conjugation can substantially improve immunoassay performance, providing an alternative to repeated immunization for assay development.

## Method details

### Chemicals and reagents

Lyophilized α-CPA was purchased from Sigma-Aldrich (Taufkirchen, Germany). Other mycotoxins used in cross-reactivity included ergotamine (Sigma-Aldrich), ergometrine (Sigma-Aldrich), penitrem A (Fermentek, Jerusalem, Israel), paxilline (ENZO Life Sciences, Lörrach, Germany), terpendole E (Abcam, Cambridge, United Kingdom, and verruculogen (Cayman Chemicals, Hamburg, Germany). The concentration of the α-CPA standard solution (1.0 mg/mL) was checked by UV-spectroscopy, using absorbance maxima at 225 (Ɛ = 39,810), 253 (Ɛ = 16,595), 275 (Ɛ = 19,054), 284 (Ɛ = 20,417) and 292 (Ɛ = 17,378) [19]. Bovine serum albumin (BSA), casein sodium salt, 3,3´,5,5´-tetramethyl benzidine (TMB) and Tween 20 were also from Sigma-Aldrich (Taufkirchen, Germany). Keyhole limpet hemocyanin (KLH) was purchased from Merck (Darmstadt, Germany). Swine anti-rabbit IgG-HRP was from DAKO (Hamburg, Germany). Methanol and all other reagents used were at least of analytical grade. Isolates of white mold were obtained from commercial soft cheeses from the German market. They were tentatively identified by MALDI-TOF-MS as *P. commune*/*P. camemberti* (including *P. fuscoglaucum, P. biforme, P. camemberti* var. *camemberti*, or *P. camemberti* var. *caseifulvum*, according to Ropars et al. [4]). These isolates were taken from a previous study [20]. Malt extract agar (MEA) was obtained from Oxoid Limited (Hampshire, United Kingdom). EIA microtiter plates were from Thermo Fisher Scientific (Waltham, USA). Solid phase antigens for plate coating were diluted with sodium bicarbonate buffer (0.05 mol/L, pH 9.6). Blocking solution was 2 % casein sodium salt in phosphate-buffered saline (PBS, 0.01 mol/L, pH 7.2), plate wash solution was distilled water containing 0.25 mL Tween 20 and 8.5 g NaCl per liter. The α-CPA toxin standard solutions were prepared in 10 % methanol/PBS. Swine anti-rabbit IgG-HRP was prepared with 1 % casein sodium salt in PBS. Enzyme substrate/chromogen solution was prepared according to Ackermann et al. [15]. Acetylated α-CPA was prepared as described by Usleber et al. [21] with slight modifications. One mg of α-CPA (in 0.5 mL pyridine) was mixed with 50 µL of acetic anhydride and incubated at 60°C for two hours. The solvent was evaporated under nitrogen flow, the residue redissolved with 0.5 mL of methanol. Further dilutions were made with 10 % methanol/PBS.

### Synthesis and characterization of immunochemicals

#### Antibody production

For preparation of polyclonal anti-α-CPA antibodies, the mycotoxin was coupled to KLH via the Mannich condensation reaction with formaldehyde [11], using reaction conditions used previously for other mycotoxins [15,17]. In brief, 3 mg of α-CPA (9 µmol) were dissolved in 300 µL dimethyl sulfoxide (DMSO) and mixed with KLH (26.7 mg (9 nmol), dissolved in 2 mL of 0.1 mol/L sodium acetate buffer [pH 4.2]). Then, 500 μL of 37 % aqueous formaldehyde solution (approximately 6.2 mmol) were added dropwise under constant stirring. The mixture was incubated at 37°C for 20 h, and then dialyzed against 3 × 6 L PBS at 4°C. Custom immunization of four rabbits and antiserum collection was done by Seramun Diagnostica GmbH, Heidesee (Germany), under licence of the responsible authority 2347-A-15-1-2018 (LVAG Brandenburg, Germany). Each animal was immunized by intracutaneous injection of 170 µL (∼1 mg of protein) α-CPA-FA-KLH, diluted with 330 µL of distilled water and then emulsified with complete Freund's adjuvant. Booster injections were given with the same amount of conjugate emulsified with incomplete Freund's adjuvant. Blood serum from one rabbit, collected 39 weeks after primary immunization, was used for further experiments.

#### Solid phase antigen production (α-CPA-BSA)

For each conjugation experiment, 1 mg α-CPA (3 µmol) was dissolved with 200 µL dimethyl sulfoxide and mixed with 9.8 mg BSA (0.15 µmol, dissolved with 1 mL of 0.1 mol/L sodium acetate buffer [pH 4.2]). Then 100 µL of aqueous formaldehyde solution 37 % (approximately 1.2 mmol) was added dropwise.

The initial α-CPA-BSA conjugate had been prepared using a reaction time of 20 h and a reaction temperature of 37°C. In a second set of experiments, eight different α-CPA-BSA conjugates were prepared with modified reaction conditions in terms of reaction temperature and time as shown in Table 1.

**Table 1:** EIA standard curve parameters obtained with α-CPA-BSA solid phase antigens prepared with formaldehyde at different reaction conditions. Values represent the mean of two tests. *For some curves, no IC_70_ was obtained because of high background absorbance.

Reaction condition		Inhibition concentration (ng/ml)			Absorbance B_0_
Temp.,°C	Time, h	IC_20_ (LOD)	IC_50_	IC_70_	
8	0.25	10 ± 1.7	130 ± 41	500 ± 230	1.7 ± 0.4
8	0.5	140 ± 52	760 ± 95	2000 ± 140	1.9 ± 0.6
8	1	50 ± 10	660 ± 80	2100 ± 260	1.8 ± 0.3
8	24	76 ± 8	870 ± 23	3400 ± 470	1.9 ± 0.24
20	2	140 ± 120	1000 ± 750	3200 ± 2000	2.0 ± 0.3
20	24	47 ± 37	2100 ± 540	-*	0.4 ± 0.03
20	72	110 ± 26	920 ± 420	2600*	0.2 ± 0.05
37	24	100 ± 80	1900 ± 140	5100*	1.1 ± 0.68

After the reaction time was completed, all conjugates were dialyzed against 3 × 6 L PBS at 4°C, and then were stored at -18°C until use. Microtiter plates were coated with each of these conjugates (100 µL per well) at a 1:500 dilution in sodium bicarbonate buffer (pH 9.6).

#### Competitive indirect EIA

Free protein binding sites of α-CPA-FA-BSA coated microtiter plates were blocked by adding 200 µL of 2 % casein sodium salt in phosphate buffered saline (PBS, pH 7.2) for 30 minutes, then the plates were washed and made semidry. Then each 50 µL per well of α-CPA standard solution (10,000-1.5 ng/mL, in 10 % methanol/PBS) and anti- α-CPA antiserum (1:1,000 in PBS, pH 7.2) was added, and incubated for 1 h at ambient temperature. The plates were washed again, swine anti-rabbit IgG HRP conjugate (in 1 % casein sodium salt/PBS) was added (100 µL per well), and incubated for 1 hour. After a final washing step, enzyme substrate/chromogen solution was added (100 µL per well). The enzyme reaction was stopped with 100 µL 1 mol/L H_2_SO_4_ per well after 15 minutes. The UV-absorbance at 450 nm was measured with a Sunrise microplate reader (Tecan, Crailsheim, Germany). The absorbance data were computed by Magellan EIA calculation software (Tecan). The absorbance values (B) of α-CPA standard concentrations were used to generate a standard curve after conversion into relative absorbance values (B/B_0_x100). The absorbance value of the blank test solution was set as B_0_. Standard curves as established for all conjugates in the competitive indirect EIA format were compared using the following parameters: inhibition concentrations IC_20_, set as the standard curve cut-off value (limit of detection, LOD); IC_50_; intra-assay coefficients of variation; non-specific background binding. Furthermore, an optimum absorbance value of B_0_ under the conditions of the EIA was considered as desirable in aspects of test robustness.

Test specificity was checked in cross-reactivity experiments using the acetylated α-CPA and a set of structurally similar mycotoxins, namely tenuazonic acid, penitrem A, paxilline, terpendole E, verruculogen, ergometrine (ergonovine), and ergotamine. These toxins were tested at concentrations up to 10 µg/mL.

Penicillium isolates were cultivated on malt extract agar (MEA) for 5 days at 25°C. Then, agar plugs of each 1 cm^2^ from areas with good fungal growth were extracted with 1 ml of methanol, as described previously by Rehagel et al. [18]. The extracts were centrifuged and the supernatant diluted 1:10 with PBS for EIA analysis, further dilutions were made with 10 % methanol/PBS if necessary.

### Method validation

To systematically evaluate the influence of conjugation parameters, reaction time and temperature were varied in a defined set of experiments, and the resulting conjugates were compared based on assay sensitivity, background signal, and dynamic range. An established in-house protocol for formaldehyde-mediated conjugation of α-CPA to KLH and BSA was used as a baseline, employing a reaction time of approximately 20 h at 37°C. This approach had previously been applied successfully to several mycotoxins containing indole or hydroxyl functionalities, including the structurally related indole diterpene penitrem A [18], and was therefore selected as a starting point.

Both the hydroxyl group and the indole nitrogen of α-CPA (Fig. 1) represent potential sites for formaldehyde-mediated conjugation; however, the exact linkage could not be resolved due to the single-step coupling to the carrier protein. UV spectroscopic analysis of α-CPA-BSA and α-CPA-KLH conjugates showed increased absorbance at 225 nm compared to unconjugated proteins, qualitatively indicating successful hapten conjugation. Due to the turbidity of the conjugate solutions, quantitative estimation of hapten loading was not feasible. Additional analytical methods such as SDS-PAGE or mass spectrometry were not performed because of limited material availability. However, successful conjugation was functionally confirmed by specific antibody binding and competitive inhibition in the EIA.

Immunization with α-CPA-KLH resulted in moderate immunogenicity. Only two out of four rabbits produced antibodies showing competitive inhibition by free α-CPA when α-CPA-BSA was used as solid phase antigen. The best-performing antiserum yielded IC_50_ values between 500 and 1000 ng/mL and IC_20_ values between 40 and 100 ng/mL. In addition, low absolute absorbance (B_0_ = 0.2-0.5) and high non-specific background absorbance (>30 %) limited assay robustness and precluded practical application. By contrast, several published EIAs for α-CPA using formaldehyde condensation reported substantially lower detection limits [[5], [6], [7], [8]]. Rather than initiating a second immunization, assay improvement was pursued by systematically modifying conjugation parameters used for preparation of the solid-phase antigen. Attempts to generate enzyme-labelled α-CPA conjugates via formaldehyde-mediated coupling to horseradish peroxidase were unsuccessful (data not shown); therefore, subsequent experiments focused on optimizing α-CPA-BSA conjugation conditions.

A series of eight α-CPA-BSA conjugates was prepared using different combinations of reaction temperature and reaction time. Due to limited availability and cost of α-CPA standard material, replicate conjugations and full factorial testing of all parameter combinations were not feasible. All conjugates specifically bound to the anti-α-CPA antibodies when used as solid-phase antigens, and binding was competitively inhibited by free α-CPA.

Although assay performance varied between conjugates (Table 1), pronounced differences in sensitivity, background absorbance, and absolute absorbance of B_0_ indicated clear trends. The lowest temperature and the shortest reaction time provided the best results. Reaction time mainly affected sensitivity, whereas temperature influenced non-specific background absorbance. All conjugates prepared at 20°C or 37°C yielded high non-specific background absorbance, limiting the measuring range and reproducibility. The largest difference between B_0_ and background absorbance was observed for conjugates prepared at 8°C, which enhances test robustness and performance.

**Fig. 2:**
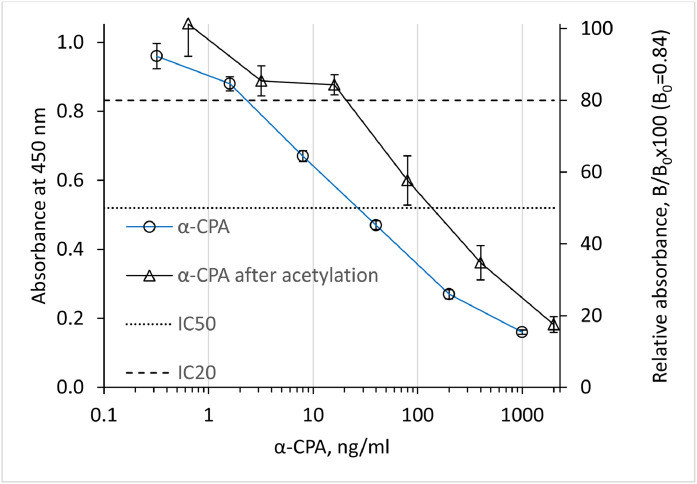
EIA standard curve of the optimized test system for α-cyclopiazonic acid, using α-CPA-BSA conjugate at 8°C with reaction time 15 min. For comparison, the standard curve established with acetylated α-cyclopiazonic acid.

The final assay was established using α-CPA-BSA prepared at 8°C with a reaction time of 15 minutes. After adjusting α-CPA standard concentrations and assay conditions to achieve B_0_ values around 1 absorbance unit, the assay produced stable and reproducible results (Fig. 2). Evaluation of ten standard curves over six weeks yielded IC_20_ = 2.4 ± 0.21 ng/mL (range 2.0-2.8 ng/mL), IC_50_ = 25 ± 3.3 ng/mL (range 17-29 ng/mL), and IC_70_ = 120 ± 18 ng/mL (range 92-140 ng/mL).

The sensitivity of the optimized assay was in the same range as reported previously [[5], [6], [7], [8], [9], [10]] and represented a substantial improvement over the initial configuration. The achieved assay sensitivity is suitable for screening purposes, such as evaluating α-CPA production in fungal cultures.

These findings highlight that conjugation conditions, often applied without optimization, can represent a critical determinant of assay performance and should be considered a key parameter in immunoassay development.

Potential cross-reactivity with structurally related α-CPA-analogues, including indole and oxindole derivatives, was considered based on previous reports [1]. However, these derivatives were not available for experimental evaluation. Several other indole-type mycotoxins, including ergonovine, ergotamine, paxilline, penitrem A, verruculogen, and terpendole E, showed no competitive inhibition at 10 µg/mL, indicating cross-reactivities below 0.01 %. Acetylated α-CPA still showed binding inhibition (Fig. 2), with IC_20_ = 24 ng/mL, IC_50_ = 130 ng/mL and IC_70_ = 580 ng/mL. These values were about 5-10 times higher than that of the parent compound, meaning a relative cross-reactivity of 10-20 %. This low cross-reactivity suggests that conjugation likely occurs at the indole group rather than the hydroxyl group. In the latter case, cross-reactivity ≥100 % would be expected, as an acetyl group at C6 (Fig. 1) would mimic a condensation site. Because acetyl- α-CPA was not purified prior to analysis, the presence of residual α-CPA cannot be excluded. Therefore, the interpretation regarding the probable conjugation site should be considered indicative rather than definitive.

**Fig. 3:**
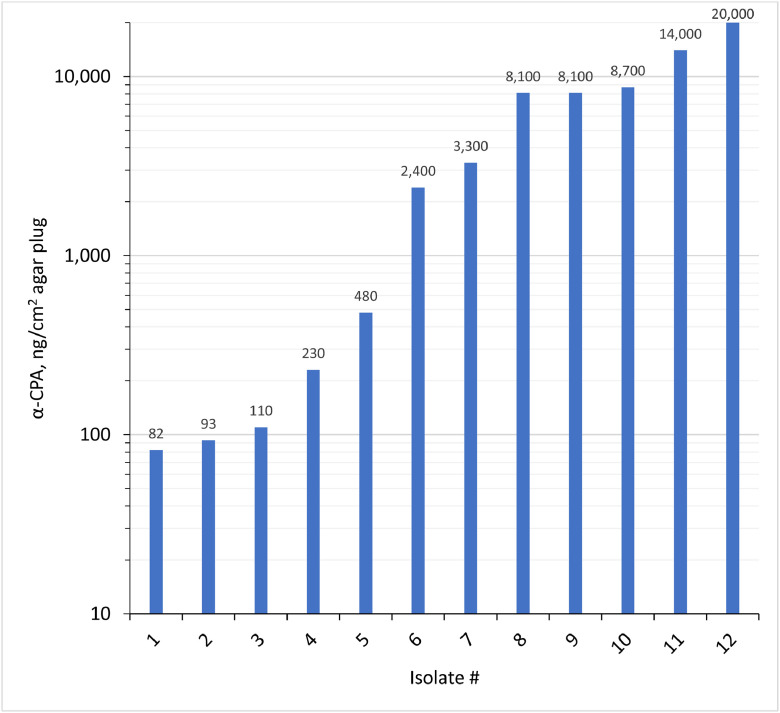
EIA results for α-CPA in 12 isolates of white mold cultured on malt extract agar (extracts of 1 cm^2^ agar plugs).

The optimized EIA was applied to fungal culture extracts from MEA agar plugs (1 cm^2^) derived from 12 different soft cheeses, tentatively identified as *Penicillium commune*/*P. camemberti* by MALDI-TOF-MS [30]. All isolates produced detectable α-CPA, with levels ranging from 0.08 µg/cm^2^ to 20 µg/cm^2^ (Fig. 3). Considerable variability between isolates was observed, highlighting the utility of the assay for comparative assessment of α-CPA production rather than absolute quantification in foods. Overall, the optimized EIA provides a rapid and practical tool for evaluating α-CPA production in fungal cultures. The approach may also be applicable to other small-molecule analytes.

Modifying reaction time and temperature during formaldehyde-mediated conjugation for α-CPA-protein conjugates substantially improved EIA sensitivity and overall performance. These findings may be applicable to other immunoassays, as optimizing existing conjugation conditions can improve assay performance without requiring additional antibody generation. Consequently, this strategy offers a practical alternative to repeated immunization, potentially reducing experimental effort. Ongoing work is investigating whether this approach can be extended to other small-molecule analytes.

## Limitations

Not applicable

Supplementary material *and/or* additional information [OPTIONAL]

## CRediT author statement

**Leon Hart:** Conceptualization, Validation, Formal analysis, Investigation, Writing – original draft. **Christina Rehagel:** Investigation, Supervision, Writing - review & editing. **Daniela Schale:** Investigation, Writing - review & editing. **Madeleine Plötz:** Funding acquisition, Writing - review & editing. **Ewald Usleber:** Funding acquisition, Project administration, Supervision, Writing - review & editing.

## Declaration of competing interest

The authors declare that they have no known competing financial interests or personal relationships that could have appeared to influence the work reported in this paper.

## Data Availability

No data was used for the research described in the article.
